# miR-34a promotes bone regeneration in irradiated bone defects by enhancing osteoblastic differentiation of mesenchymal stromal cells in rats

**DOI:** 10.1186/s13287-019-1285-y

**Published:** 2019-06-18

**Authors:** Huan Liu, Yan Dong, Xiaoke Feng, Liya Li, Yang Jiao, Shizhu Bai, Zhihong Feng, Hao Yu, Xuejian Li, Yimin Zhao

**Affiliations:** 1 State Key Laboratory of Military Stomatology & National Clinical Research Center for Oral Diseases & Shaanxi Key Laboratory of Stomatology, Department of Prosthodontics, School of Stomatology, The Fourth Military Medical University, No. 145 West Changle Road, Xi’an, 710032 China; 2 Xi’an Institute of Tissue Engineering and Regenerative Medicine, No. 169 West Changle Road, Xi’an, 710032 China; 3 Department of Stomatology, The 7th Medical Center of PLA General Hospital, NO.5, Nanmencang, Dongsishitiao Street, Beijing, 100700 China

**Keywords:** miR-34a, Bone marrow mesenchymal stromal cell, Bone regeneration, Osteoblastic differentiation, Radiotherapy

## Abstract

**Background:**

Radiation exposure negatively affects the regenerative ability and makes reconstruction of bone defects after tumor section difficult. miR-34a is involved in radiation biology and bone metabolism. The aim of this study was to investigate whether miR-34a could contribute to bone regeneration in irradiated bone defects.

**Methods:**

The expression of miR-34a was analyzed during the osteoblastic differentiation of irradiated BMSCs and bone formation in irradiated bone defects. miR-34a mimics and miR-34a inhibitor were used to upregulate or suppress the expression of miR-34a in BMSCs irradiated with 2 or 4 Gy X-ray radiation. In vitro osteogenesis and subcutaneous osteogenesis were used to assess the effects of miR-34a on the osteogenic ability of radiation-impaired BMSCs. Collagen-based hydrogel containing agomiR-34a or antagomiR-34a were placed into the 3-mm defects of irradiated rat tibias to test the effect of miR-34a on bone defect healing after irradiation.

**Results:**

miR-34a was upregulated in the process of bone formation after irradiation. Transfecting radiation-impaired BMSCs with miR-34a mimics enhanced their osteoblastic differentiation in vitro by targeting NOTCH1. Overexpression of miR-34a enhanced the ectopic bone formation of irradiated BMSCs. In situ delivery of miR-34a promoted bone regeneration in irradiated bone defects.

**Conclusions:**

miR-34a promoted the osteoblastic differentiation of BMSCs and enhanced the ectopic bone formation after irradiation. miR-34a promoted bone defect healing in irradiated rat tibias. miR-34a-targeted therapy might be a promising strategy for promoting the reconstruction of bone defects after radiotherapy.

**Electronic supplementary material:**

The online version of this article (10.1186/s13287-019-1285-y) contains supplementary material, which is available to authorized users.

## Background

Surgical procedures and radiotherapy are critical components of the multidisciplinary management of bone and soft tissue tumors [1]. The resection of tumors can result in extensive bone defects, and reconstruction of the defect is needed [2]. However, radiotherapy hampers wound healing during bone reconstruction and restoration [3], which lead to higher rates of flap losses [4] and implant failures [5] in patients with radiotherapy.

While destroying potential residual malignant cells, radiotherapy also damages adjacent normal tissues [6]. The hypocellularity, hypovascularity, and hypoxia condition after irradiation inhibits bone growth and healing [7]. In irradiated bone, fewer osteoblast cells and osteocytes are observed, which impedes osteogenesis and bone mineralization [6]. Bone marrow mesenchymal stromal cells (BMSCs) are important source of osteoblasts, but the osteoblastic differentiation of BMSCs is impaired by irradiation [8, 9]. It is necessary to discover the molecular mechanisms regulating osteogenic differentiation of BMSCs under irradiated conditions.

miR-34a is known as a tumor suppressor that represses the invasion, metastasis, and drug resistance of malignant cells [10]. miR-34a is also involved in tissue development and non-cancerous diseases [11]. It is reported that miR-34a plays a role in regulating osteogenic differentiation of stem cells and bone metabolism [12–14]. Irradiation increases the expression of miR-34a in malignant cell lines and normal cells or tissues [15]. However, the function of miR-34a in bone formation after irradiation is unknown.

In this study, we observed that miR-34a was upregulated during the osteoblastic differentiation of irradiated BMSCs and bone formation in irradiated bone defects. Overexpression of miR-34a improved the osteoblastic differentiation of irradiated BMSCs and promoted bone regeneration in bone defects of irradiated rat tibias. miR-34a exerted this effect by repressing NOTCH1. Our findings indicated that miR-34a might be a promising therapeutic target for promoting the reconstruction of the bone defect after malignant tumor resection.

## Methods

### Ethics statement

All animal experiments were approved by the Animal Care Committee of Fourth Military Medical University (Permit Number: kq-022), and all the experimental procedures were conducted following the relevant guidelines and regulations. Sprague-Dawley rats of 2 weeks old or irradiated rats were used for the isolation of BMSCs. Six female nude mice of 6 weeks old were used in the ectopic osteogenesis experiment. Male Sprague-Dawley rats weighing 240–270 g were used for irradiation. Eighteen male Sprague-Dawley rats were used for comparison of miRNA expression and bone regeneration in irradiated and non-irradiated bones. Twelve male Sprague-Dawley rats were used to study the enhancement of miR-34a on bone repair in irradiated bones.

### Culture of rat BMSCs and osteoblastic differentiation

Rat BMSCs were isolated and cultured as described before [16]. Briefly, bone marrow was flushed from tibias and femurs after all soft tissues were removed. The bone marrow suspension was fractionated by density gradient centrifugation (Histopaque-1083, Sigma, MO, USA) for 25 min at 400 g, and the mononuclear cells were used. Cells were cultured in α-minimum essential medium (α-MEM) supplemented with 10% fetal bovine serum (FBS; Sijiqing, Hangzhou, China) and 1% penicillin and streptomycin. Cells of passage 2 were used in experiments. Sprague-Dawley rats of 2 weeks old were used for the isolation of BMSCs for in vitro test and subcutaneous osteogenesis. BMSCs from irradiated rats were isolated 8 weeks after irradiation.

For osteogenic differentiation, 1 × 10^5^/well BMSCs were seeded in 12-well plates. Cells were cultured in an osteogenic medium containing 50 μg/ml ascorbic acid, 10 mM β-glycerophosphate, and 10 nM dexamethasone. Alkaline phosphatase (ALP) staining was tested by BCIP/NBT ALP color development kit (Beyotime, Shanghai, China), and intracellular ALP activity was tested by ALP assay kit (Nanjing Jiancheng Bioengineering Institute, Nanjing, China) according to the manufacturer’s protocol after induction for 7 days. Extracellular matrix mineralized nodules were tested by 1 wt% Alizarin Red S staining (Leagene Biotechnology, Beijing, China) after induction for 21 days. Alizarin Red S staining was further quantitatively analyzed by being dissolved in 10% cetylpyridinium chloride. The absorbance values were measured at 560 nm. Osteogenesis related mRNA and protein expression were tested after 14 days of osteogenic induction by quantitative real-time reverse transcription-polymerase chain reaction (qRT-PCR) and Western blot.

### Irradiation of BMSCs

When BMSCs reached 95% confluence, cells were irradiated with 0, 2, 4, and 8 Gy of X-ray radiation delivered at a rate of 1.1 Gy/minute (RS-2000 XE Biological Irradiator, Rad Source Technologies, GA, USA). The culture medium was replaced by the osteogenic medium after irradiation.

### Transfection

The BMSCs were transfected with miR-34a mimics (50 nM), mimics control (50 nM), miR-34a inhibitor (100 nM), inhibitor control (100 nM), small interfering RNA (siRNA) targeting *Notch1* mRNA (50 nM), or negative control (50 nM) (Ruibo, Guangdong, China) using Lipofectamine 2000 after irradiated by 2 or 4 Gy. The medium was replaced by the osteogenic medium 6 h after transfection.

### RNA extraction and quantitative real-time PCR (qRT-PCR)

Total RNA was extracted by TriZol (Invitrogen, CA, USA), and 500 ng total RNA was transcribed into cDNA by a PrimeScript RT reagent kit (TaKaRa, Kyoto, Japan). qRT-PCR was performed with SYBR PremixExTaq™II (TaKaRa) on the CFX96™Real Time RT-PCR System. Relative expression was calculated by the ΔΔCt method, and *Gapdh* was used for normalization. The primers were synthesized as shown in Table 1. For miRNA quantification, Bulge-loopTM qRT-PCR Primer Sets (one PT primer and a pair of qPCR primers for each set) specific for miR-34a and U6 were designed by Ruibo.

**Table 1:** Primers used for qRT-PCR

Gene	Forward primer sequence(5′-3′)	Reverse primer sequence(5′-3′)
*Runx2*	5′-AGA CCA GCA GCA CTC CAT AT-3′	5′-CTC ATC CAT TCT GCC GCT AGA-3′
*Alp*	5′-ATG GCT CAC CTG CTT CAC G-3′	5′-TCA GAA CAG GGT GCG TAG G-3′
*Ocn*	5′-AGG GCA GTA AGG TGG TGA AT-3′	5′-GCA TTA ACC AAC ACG GGG TA-3′
*Col-1*	5′-GCC TCC CAG AAC ATC ACC TA-3′	5′-GCA GGG ACT TCT TGA GGT TG-3′
*Gapdh*	5′-GGCACAGTCAAGGCTGAGAATG-3′	5′-ATGGTGGTGAAGACGCCAGTA-3′

### Western blot analysis

Cells were lysed in RIPA buffer containing a protease inhibitor cocktail (Sigma, MO, USA). Protein concentrations were quantified by the BCA protein assay (Beyotime). Forty micrograms of protein of each sample was loaded on 10% SDS-PAGE and transferred to the PVDF membranes after separation. The membranes were blocked with 5% BSA for 2h and incubated with primary antibodies for rat COL-1(Protein tech, 14695-1-AP), RUNX2 (Santa Cruz Biotechnology, sc-10758), ALP (Protein tech, 11187-1-AP), osteocalcin (OCN; Santa Cruz Biotechnology, sc-390877), NOTCH1 (Cell Signaling Technology, #4380), and GAPDH (Abcam, ab8245) overnight at 4 °C. The membranes were incubated for 2 h with secondary antibodies (Cowin Biotech, China). The protein bands were incubated with a chemiluminescence kit (Amersham Biosciences, USA) and visualized by the imaging system (Tanon 5500, China). The exposure time is 20 s for GAPDH and 100–200 s for other protein bands depending on the obtained signal intensity. The gray value of the protein bands was quantified by using Image-Pro Plus 6.0 software and normalized to that of GAPDH before comparison.

### Subcutaneous osteogenesis model

For subcutaneous osteogenesis test, 2 × 10^5^/well BMSCs were seeded in six-well plates and treated with growing medium containing 50 μg/ml vitamin C for 5 days. BMSCs were irradiated with 2 Gy and 4 Gy of X-ray radiation and transfected with miR-34a mimics, mimics control, miR-34a inhibitor, and inhibitor control as described above. The medium was replaced by osteogenic medium 6 h after transfection and further cultured for 3 days. The cultured cells along with their deposited extracellular matrix (ECM) were detached as intact sheets from the dishes (BMSC-sheets). Four layers of BMSC-sheets were composited with three layers of bone substitute (20 mg in total, particles 0.25-1 mm, Geistlich) and packaged as a block mass for subcutaneous transplantation as previously described [17]. The implants composed of cell sheets and bone substitute were subcutaneously transplanted into the backs of nude mice (*n* = 3). Samples were harvested 8 weeks after implantation for histological assay. The implants were fixed by 4% paraformaldehyde for 48 h and decalcified with 18% EDTA for 4 weeks and prepared for H&E staining.

### Irradiation of rats

Rats were fixed in a perspex jig with their tibias extended laterally. The tibias of rats were subjected to irradiation, and other parts of the body were protected with lead shielding. A single dose of 15 Gy of X-ray radiation was delivered at a rate of 1.1 Gy/minute (RS-2000 XE Biological Irradiator, Rad Source Technologies).

### Rat tibial defect model

For comparison of miR-34a expression and bone regeneration in irradiated and non-irradiated bones, the right tibia of each rat was irradiated. Bone defect surgeries were conducted 3 weeks after irradiation. A 3-mm tibial defect was generated in both tibias. Rats were sacrificed at 2, 4, and 8 weeks after surgery, and the tibias were harvested. For the test of miR-34a expression, the newly formed bone in the defect area was harvested and ground in liquid nitrogen (*n* = 3). The total RNA was extracted with Trizol reagent (Invitrogen), and miRNA expression was evaluated by qRT-PCR. Newly formed bone was evaluated by Micro-CT (Y.Cheetah, Y.XLON, Hamburg, Germany) and H&E staining (*n* = 3).

To study the enhancement of miR-34a on bone repair in irradiated bone defects, we subjected both tibias of each rat to irradiation. A 3-mm tibial defect was generated in both tibias. Two hundred fifty picomoles of agomir-34a and antagomir-34a, or equal amounts of their respective negative controls (3 μl), were dispensed into 15 μl Col-Tgel component A and 1.5 μl component B (Bioruo, Beijing, China), and then were implanted within the defects. At 8 weeks post-implantation, bone regeneration in the defect was evaluated using Micro-CT (Y.Cheetah, Y.XLON) and histological techniques as described below.

### Test of micro-CT

Tibias were harvested and fixed in 4% paraformaldehyde and scanned by Micro-CT (Y.Cheetah, Y.XLON). Scan settings were rotation of 360°, rotation step of 0.5°, kilovoltage of 80 kV, current of 62 μA, and scanning resolution of 18 μm. VG StudioMAX (Volume Graphics, Heidelberg, Germany) was used for reconstructing 3D images and data analysis. The original bone defect area (L, 2 mm; φ, 3 mm) was defined as the region of interest (ROI). The percentages of BV/TV were calculated to compare the bone regeneration (*n* = 5).

### H&E staining

Tibias were decalcified for 4 weeks in 18% EDTA (pH 7.0) and embedded in paraffin. Sections (5 µm thickness) were cut and stained with H&E according to standard protocols.

### Sequential fluorescent labelling assay

To assess bone formation and mineralization in bone defect area, different fluorochromes were injected intramuscularly at a sequence of 30 mg/kg Alizarin Red S (Sigma), 20 mg/kg Calcein (Sigma), and 20 mg/kg Tetracycline Hydrochloride (Sigma) at 3, 5, and 7 weeks after the operation. Tibias were embedded in polymethylmethacrylate (PMMA) and cut into 50-μm sections (LEICA SP1600, Wetzlar, Germany). The undecalcified sections were observed using a confocal laser scanning microscope (OLYMPUS FV1000, Tokyo, Japan), and the area of fluorescent labelling was quantified by Image-Pro Plus 6.0 software (*n* = 3).

### Statistical analysis

Results were displayed as mean ± standard deviation at least three biological replicates. Differences between two groups were determined by the Student’s *t* test. Differences among groups were analyzed by one-way ANOVA followed by Tukey’s post-test. GraphPad Prism7 software was used, and statistical significance was considered when *p* < 0.05.

## Results

### The expression of miR-34a increased in irradiated BMSCs after osteoblastic differentiation

**Fig. 1:**
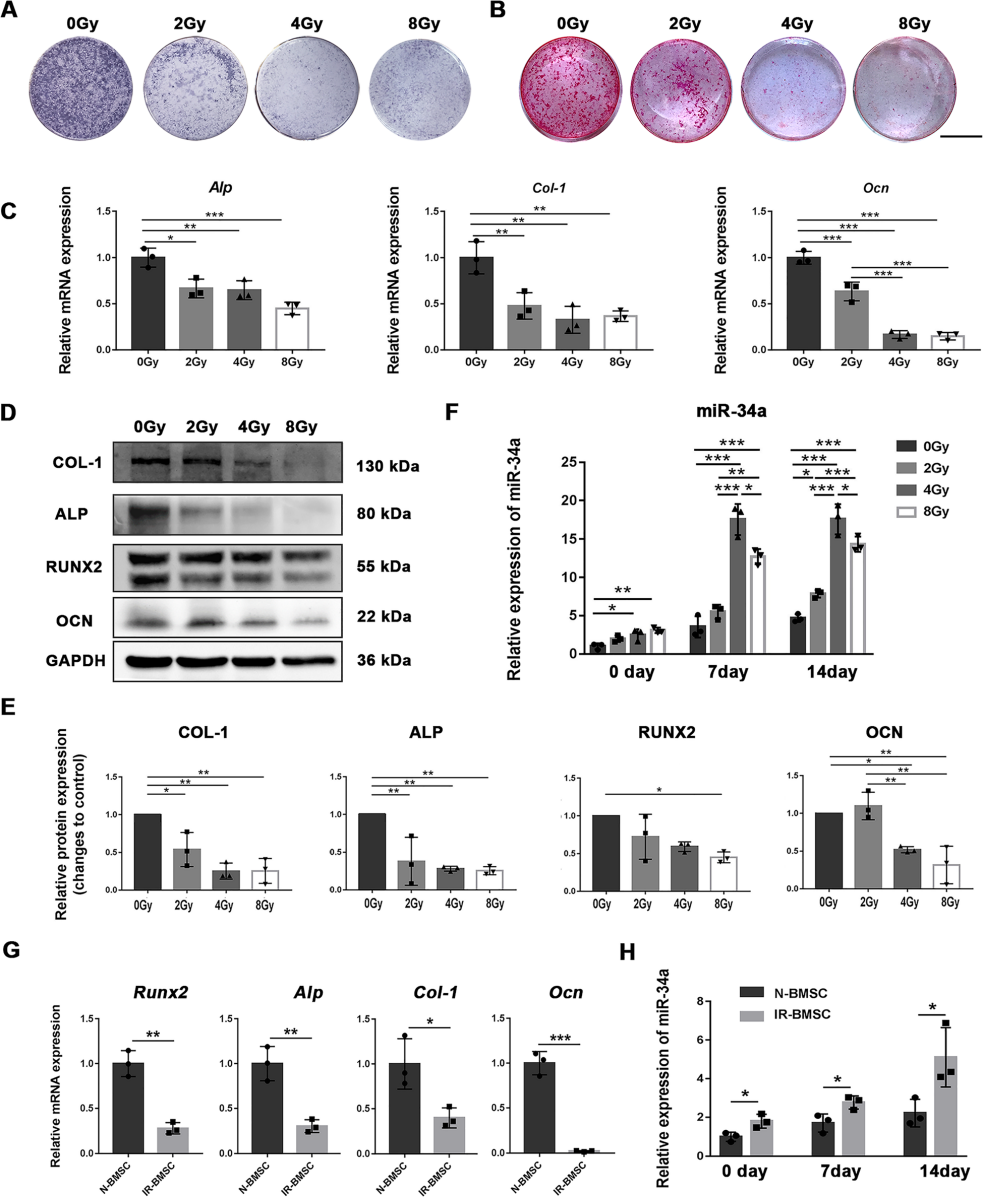
The osteoblastic differentiation and miR-34a expression of irradiated BMSCs. **a** ALP staining after osteogenic induction for 7 days. **b** Alizarin red staining after osteogenic induction for 21 days, scale bar = 1 cm. **c** Gene expression of *Runx2*, *Alp*, *Col-1*, and *Ocn* after 14 days of osteogenic induction. **d** Western blot analysis of RUNX2, ALP, COL-1, OCN, and GAPDH after 14 days of osteogenic induction. **e** The quantitative analysis of the Western blot results relative to GAPDH (fold to control). **f** Expression of miR-34a during osteoblastic differentiation. **g** Gene expression of *Runx2*, *Alp*, *Col-1*, and *Ocn* after 14 days of osteogenic induction in the BMSCs isolated from non-irradiated (N-BMSC) and irradiated tibias (IR-BMSC). **h** Expression of miR-34a of N-BMSC and IR-BMSC during osteogenic differentiation. Data are shown as mean ± SD, *n* = 3; *p < 0.05, **p < 0.01, ***p < 0.001

To verify whether miR-34a is involved in osteoblastic differentiation of radiation-impaired BMSCs, we subjected BMSCs to different doses of X-ray radiation and tested the osteogenesis and the expression of miR-34a. The cultured BMSCs showed typical BMSC characterizations (Additional file 1: Figure S1). X-ray radiation inhibited the osteoblastic differentiation of BMSCs. Both Alp (Fig. 1a) and Alizarin Red S (Fig. 1b) staining were decreased after irradiation. Irradiation caused a reduction in mRNA expression of *Alp*, *Col-1*, and *Ocn* (Fig. 1c). The protein levels of ALP and COL-1 were decreased in irradiated cell. RUNX2 protein levels of the 0 Gy group were higher than the 8 Gy group. OCN protein levels of the 0 Gy and 2 Gy group were higher than 4 Gy and 8 Gy group. (Fig. 1d, e). The expression of miR-34a was increased in the 4 Gy and 8 Gy group 24 h post-irradiation (0 day after osteogenic induction) compared to the 0 Gy group. The expression of miR-34a was higher in the 4 Gy and 8 Gy group than 0 Gy group 7 days after osteoblastic differentiation. The expression of miR-34a was higher in all the irradiated groups than 0 Gy group 14 days after osteoblastic differentiation (Fig. 1f).

We also tested the osteogenesis and the miR-34a expression in BMSCs isolated from irradiated (IR-BMSC) and non-irradiated (N-BMSC) rat tibias. IR-BMSC showed lower expression of *Runx2*, *Alp*, *Col-1*, and *Ocn* than N-BMSC 14 days after osteogenic induction (Fig. 1g). The IR-BMSC exhibited higher miR-34a level than N-BMSC after osteoblastic differentiation (Fig. 1h).

### The expression of miR-34a increased in newly formed bone after irradiation

**Fig. 2:**
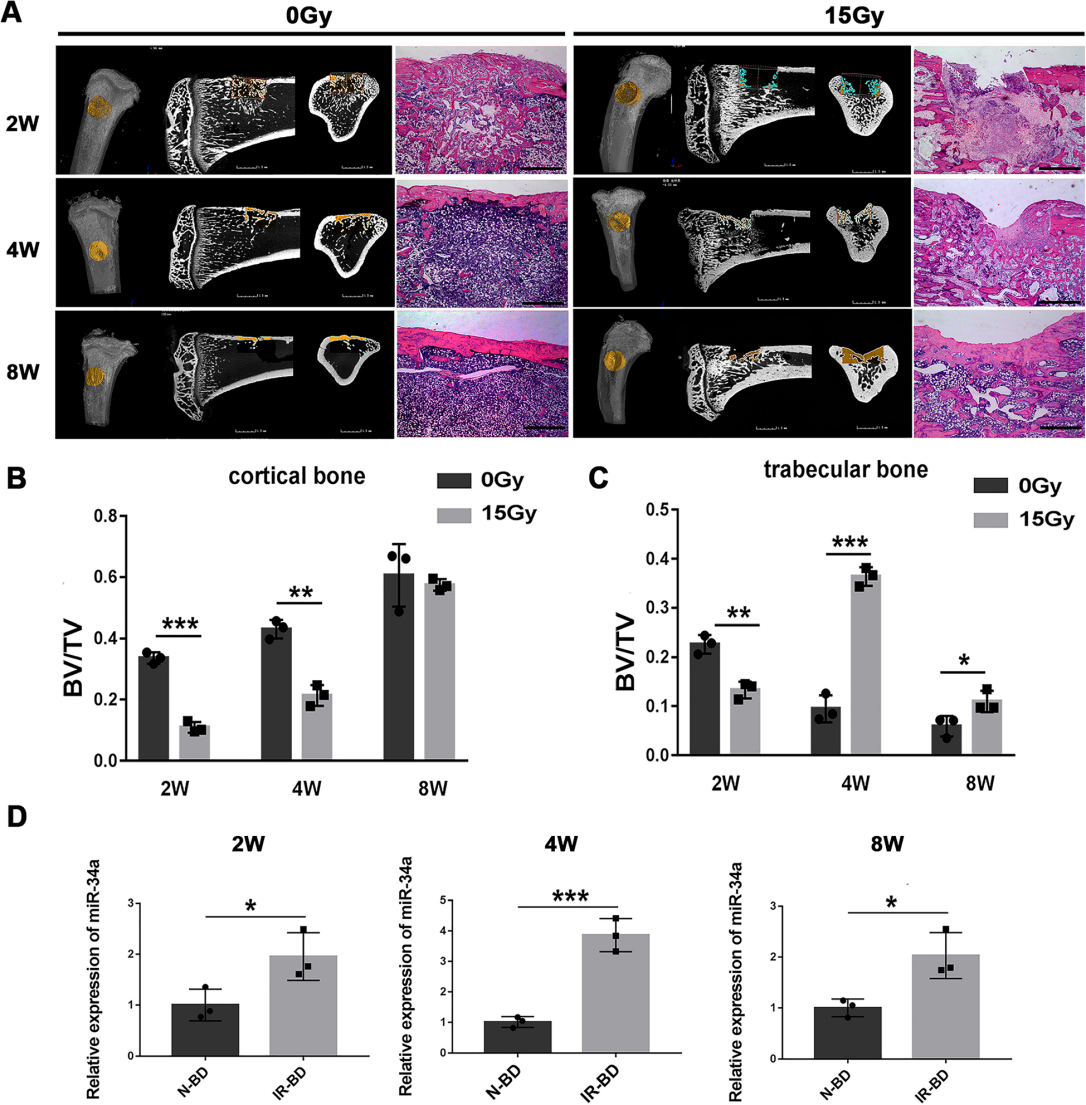
Bone formation in irradiated bone defects and the expression of miR-34a in newly formed bone. **a**, **b** Micro-CT and H&E images of bone formation in bone defects of non-irradiated (0 Gy) and irradiated (15 Gy) rats 2, 4, and 8 weeks after surgery; scale bar of CT 2D images = 1.5 mm; scale bar of H&E images = 1 mm. **d** BV/TV in the cortical bone area. **c** BV/TV in the trabecular bone area. **d** Expression of miR-34a in the newly formed bone of non-irradiated (N-BD) or irradiated (IR-BD) rats. Data are shown as mean ± SD, *n* = 3; *p < 0.05. **p < 0.01, ***p < 0.001

Since miR-34a level was higher in the irradiated BMSCs than the non-irradiated BMSCs after osteoblastic differentiation in vitro, we further investigated whether miR-34a was involved in bone formation in vivo after irradiation. Irradiation inhibited bone formation in the defect area (Fig. 2a). The primary callus formation was postponed by irradiation, as the BV/TV of cortical and trabecular bone were lower in the irradiated group than the non-irradiated group at 2 weeks (Fig. 2b, c). Irradiation caused a delay in the osseous closure, as the newly formed cortical bone was less in the irradiated group than in the non-irradiated group at 4 weeks (Fig. 2b). Subsequent remolding was also reduced in the irradiated group, as the BV/TV of trabecular bone was higher in the irradiated group than the non-irradiated group at 4 and 8 weeks (Fig. 2c). The expression level of miR-34a in the newly formed bone was higher in the irradiated group than the non-irradiated group at 2, 4, and 8 weeks after bone defect surgery (Fig. 2d).

### miR-34a overexpression enhanced the osteoblastic differentiation of irradiated BMSCs in vitro

To evaluate the effect of miR-34a on osteoblastic differentiation of irradiated BMSCs, we transfected 2 Gy or 4 Gy irradiated BMSCs with miR-34a mimics, miR-34a inhibitor or their controls. The transfection efficiency was estimated to be 75–90%. Two days after transfection, miR-34a showed a > 150-fold increase in the miR-34a mimics group and > 38% reduction in the miR-34a inhibitor group compared with their negative controls. The expression levels remained an 80-fold increase in the miR-34a mimics group and > 30% reduction in the inhibitor group compared with their negative controls after 14 days of osteogenic induction (Additional file 1: Figure S2).

**Fig. 3:**
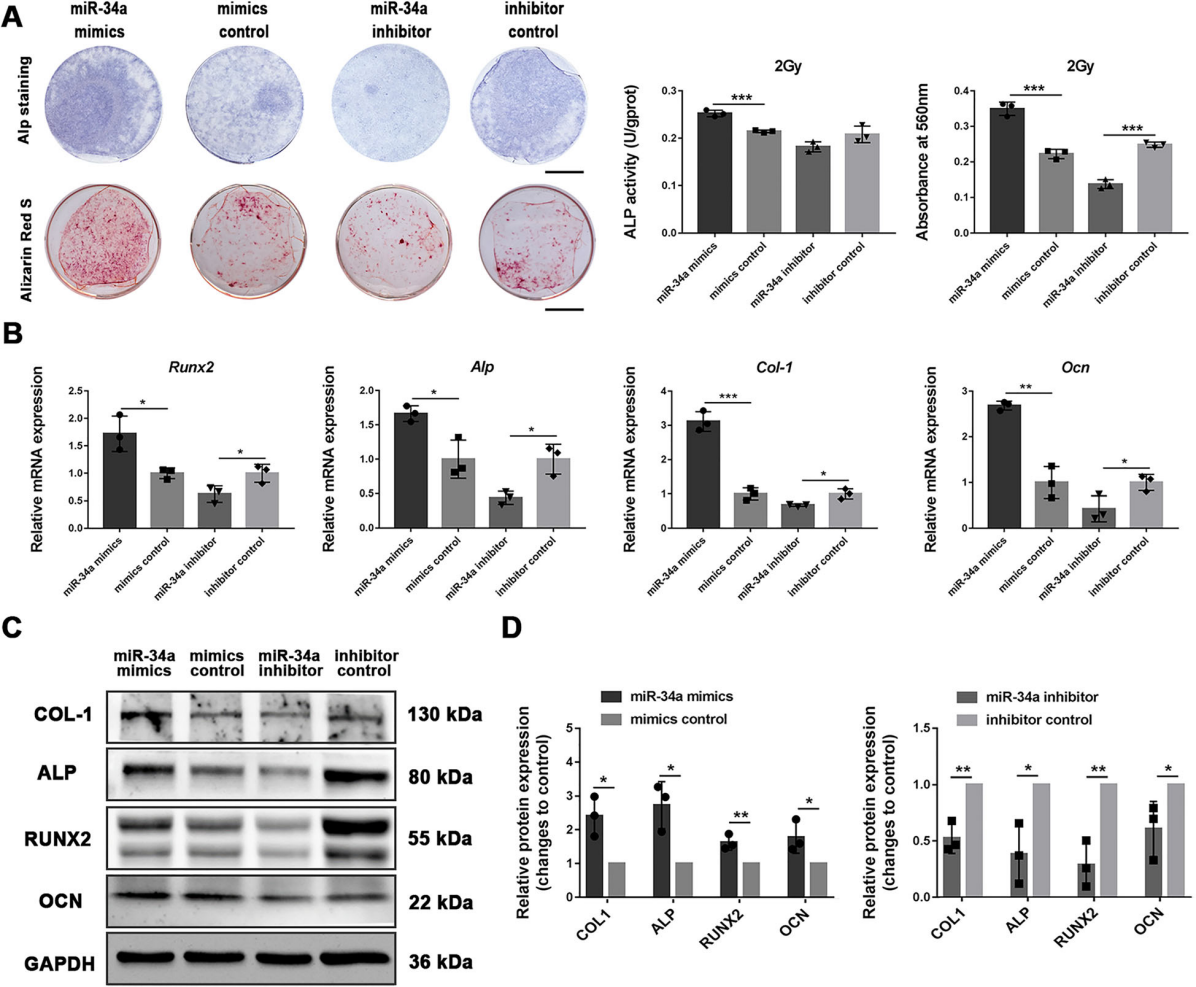
miR-34a overexpression enhanced the osteoblastic differentiation of 2 Gy irradiated BMSCs in vitro. **a** ALP staining, intracellular ALP activity, Alizarin red staining, and the quantitative colorimetric results of Alizarin red staining for BMSCs irradiated with 2 Gy after osteogenic induction; scale bar = 1 cm. **b** Gene expression of *Runx2*, *Alp*, *Col-1*, and *Ocn* after 14 days of osteogenic induction. **c** Western blot analysis of RUNX2, ALP, COL-1, OCN, and GAPDH after 14 days of osteogenic induction. **d** The quantitative analysis of the Western blot results relative to GAPDH (fold to control). Data are shown as mean ± SD, *n* = 3; *p < 0.05, **p < 0.01, ***p < 0.001

For 2 Gy irradiated BMSCs, Alp staining and alizarin red staining were increased in the miR-34a mimics group and decreased in the miR-34a inhibitor group compared with their control groups (Fig. 3a). The Alp activity was enhanced in the miR-34a mimics group compared with the control group (Fig. 3a). The expression of RUNX2, ALP, COL-1, and OCN at both mRNA and protein levels were enhanced by miR-34a overexpression and reduced by miR-34a suppression (Fig. 3b–d). Similar tendency was found in the 4 Gy irradiated BMSCs (Additional file 1: Figure S3).

### miR-34a regulated the osteoblastic differentiation of irradiated BMSCs by repressing NOTCH1

**Fig. 4:**
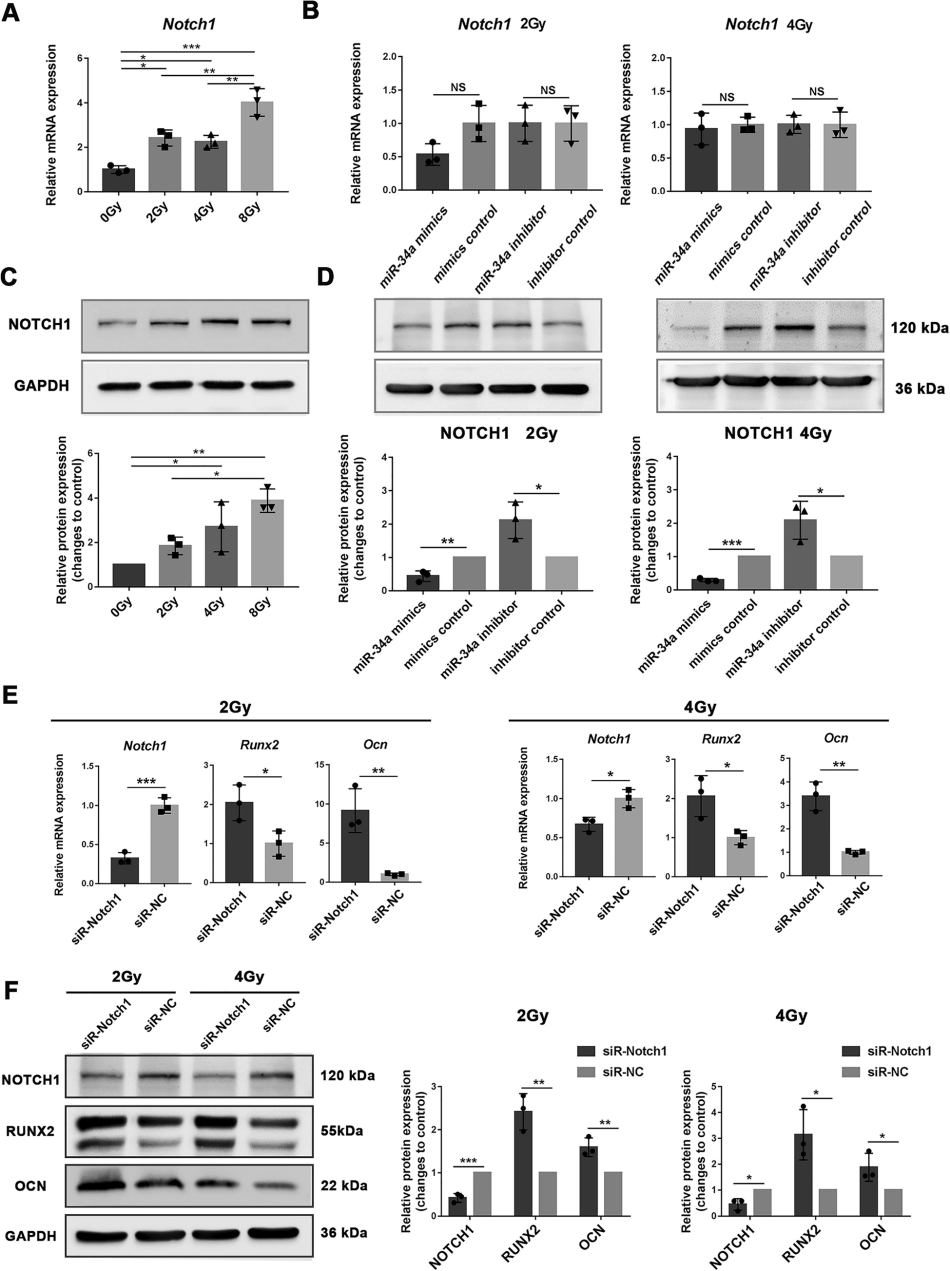
miR-34a regulated the osteoblastic differentiation of irradiated BMSCs by suppressing NOTCH1. qRT-PCR (**a**) and Western blot (**c**) analysis of NOTCH1 in irradiated BMSCs after 14 days of osteogenic induction. qRT-PCR (**b**) and Western blot (**d**) analysis of NOTCH1 after 14 days of osteogenic induction for BMSCs irradiated with 2 Gy and 4 Gy. qRT-PCR (**e**) and Western blot (**f**) analysis of NOTCH1, RUNX2 and OCN in irradiated BMSCs after NOTCH1 knockdown and 14 days of osteogenic induction. Data are shown as mean ± SD, *n* = 3; *p < 0.05, **p < 0.01, ***p < 0.001

Notch signaling in bone marrow suppresses the osteoblast differentiation of BMSCs [18]. NOTCH1 was previously identified as a direct target of miR-34a in tumor cells [19, 20]. The relationship between miR-34a and NOTCH1 was previously confirmed in human adipose-derived stem cells during osteoblastic differentiation process [14]. To confirm whether miR-34a promotes osteoblastic differentiation of irradiated BMSCs through regulating NOTCH1, we tested the expression of NOTCH1 in irradiated BMSCs after osteogenic induction. The mRNA expression of *Notch1* of 2, 4, and 8 Gy irradiated BMSCs was higher than the non-irradiated BMSCs (Fig. 4a). The protein expression of NOTCH1 was higher in the 4 Gy and 8 Gy group than in the 0 Gy group. NOTCH1 protein expression was higher in the 8 Gy group than in the 2 Gy group (Fig. 4c). NOTCH1 protein expression was downregulated in the miR-34a mimics group and upregulated in the miR-34a inhibitor group compared with their negative controls (Fig. 4d). *Notch1* mRNA expression did not show this trend (Fig. 4b).

To further confirm the interaction between NOTCH1 and the osteoblastic differentiation of BMSCs after irradiation, we transfected irradiated BMSCs with siR-Notch. The transfection efficiency was about 80%, and *Notch1* mRNA expression was reduced by siR-Notch1 2 days after transfection (Additional file 1: Figure S4). After osteogenic induction, the NOTCH1 expression remained decreased in siR-Notch1 groups compared with the control groups (Fig. 4e). Downregulation of NOTCH1 enhanced the mRNA and protein expression of RUNX2 and OCN (Fig. 4e, f). Taken together, these data indicated that miR-34a played a role in regulating osteogenesis under irradiated conditions through NOTCH1.

### miR-34a overexpression enhanced the ectopic bone formation of irradiated BMSCs

**Fig. 5:**
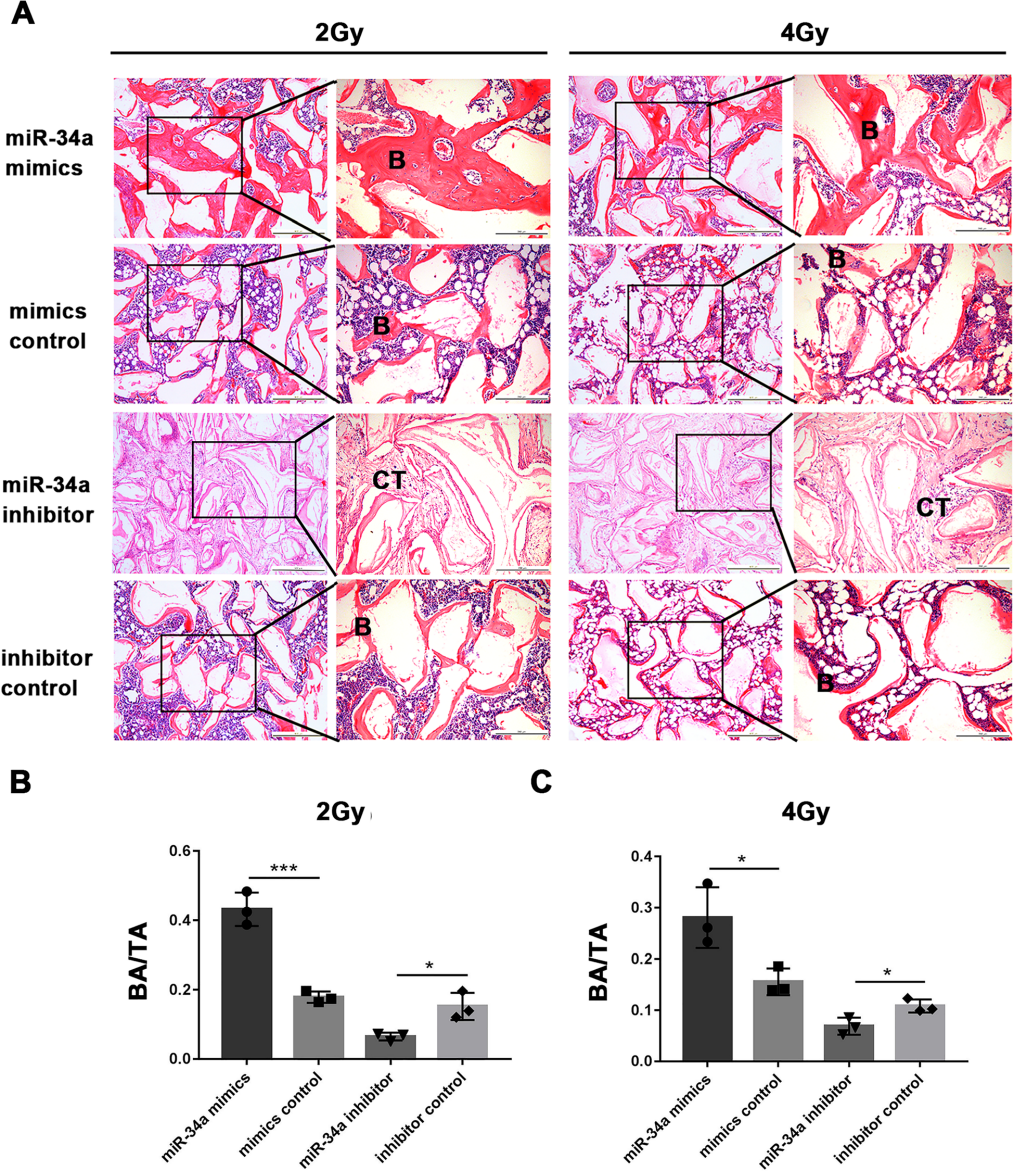
miR-34a overexpression enhanced the ectopic bone formation of irradiated BMSCs. **a** Representative H&E staining of ectopic bone formation of BMSCs irradiated with 2 Gy or 4 Gy (B, bone; CT, connected tissue); scale bar of images on the left, 500 μm; scale bar of images on the right, 200 μm. **b**, **c** The percentages of new bone area to total area (BA/TA) of BMSCs irradiated with 2 Gy or 4 Gy. Data are shown as mean ± SD, *n* = 3; *p < 0.05, **p < 0.01, ***p < 0.001

To investigate whether miR-34a could improve the ectopic bone formation of irradiated BMSCs, BMSCs irradiated with 2 Gy or 4 Gy were transplanted subcutaneously into nude mice for 8 weeks after transfected with miR-34a mimics, miR-34a inhibitor, or their negative controls. H&E staining showed that bone formation was enhanced by miR-34a overexpression (Fig. 5a). The percentage of bone area to total area (BA/TA) was used to quantify and compare the amount of newly formed bone. BA/TA was increased by miR-34a overexpression and decreased by miR-34a suppression (Fig. 5b, c).

### miR-34a overexpression enhanced bone formation in irradiated bone defects

**Fig. 6:**
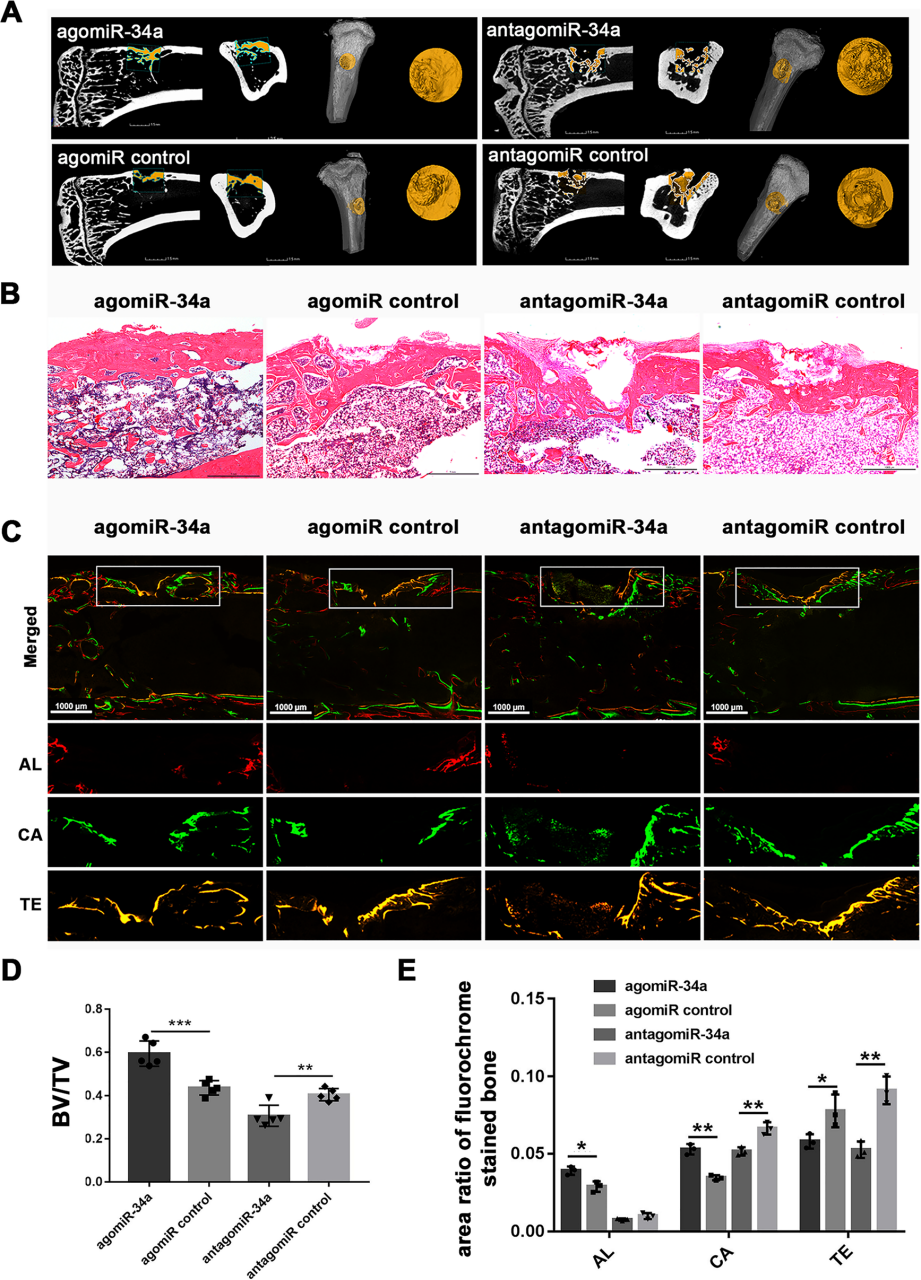
miR-34a overexpression enhanced bone formation in irradiated bone defects. **a** 2D and 3D micro-CT images of bone formation in the defect area of irradiated rats at 8 weeks; scale bar of CT 2D images = 1.5 mm. **b** Representative H&E staining of new bone formation; scale bar = 1 mm. **c** Sequential fluorescent labeling of bone formation and mineralization; Alizarin Red S (AL), Calcein (CA), Tetracycline Hydrochloride (TE); scale bar = 1 mm. **d** The morphometric analysis of BV/TV for the micro-CT images (*n* = 5). **e** The quantitative results of sequential fluorescent labeling (*n* = 3). Data are shown as mean ± SD; *p < 0.05, **p < 0.01, ***p < 0.001

To determine whether miR-34a could enhance bone formation in irradiated bone defects, we established a 3-mm tibial bone defect model 3 weeks after irradiation. The collagen-based hydrogel containing agomir-34a, antagomir-34a, or equal amounts of their respective negative controls were placed into the defect sites. New bone formation in the defect was assessed by micro-CT 8 weeks after surgery. More regenerated bone was observed in the agomiR-34a group compared with the negative control group (Fig. 6a). BV/TV was higher in the agomiR-34a group than the negative control group (Fig. 6d). The antagomiR-34a group showed less regenerated bone and decreased BV/TV compared with the negative control group (Fig. 6a, d). HE staining (Fig. 6b) confirmed the results of the micro-CT scanning.

Sequential fluorescent labelling was used to measure bone mineralization and deposition during bone defects repair (Fig. 6c), and the labelling area was quantified (Fig. 6e). Alizarin Red S and Calcein labelling area in the agomiR-34a group were larger than in the agomiR control group, while Tetracycline Hydrochloride area in the agomiR-34a group was less than in the agomiR control group. These results indicated that bone formation and subsequent remolding was enhanced by agomiR-34a. Calcein labelling and Tetracycline Hydrochloride area in the antagomiR-34a group were less than in the agomiR control group, indicating that bone defect repair in irradiated tibias was delayed by antagomiR-34a.

## Discussion

The inhibitory effects of radiotherapy on bone formation hinder the reconstruction of bone defects after the resection of bone and soft tissue tumors. In this study, we found a novel strategy based on miRNA to promote bone formation after irradiation. We identified that miR-34a was upregulated during the process of osteogenesis after irradiation. We showed that overexpression of miR-34a promoted the osteoblastic differentiation of irradiated BMSCs. Based on ectopic osteogenesis and tibial defect model, we certified that miR-34a overexpression improved bone regeneration under irradiated conditions.

An increasing number of researches have focused on the osteogenesis and miRNA function, both under the biological and pathological conditions. miR-33-5p is a key factor in the development of osteopenia induced by mechanical unloading, and miR-33-5p-targeted treatment could alleviate the osteopenia [21]. miR-26a was negatively correlated with bone loss in osteoporotic mice, and miR-26a could be used to restore the osteogenic capacity MSCs under osteoporotic circumstance [22]. However, the role of miRNAs in regulating bone formation after irradiation is poorly understood. We found that miR-34a was expressed at a higher level in irradiated BMSCs compared with non-irradiated BMSCs after osteogenic induction. The expression of miR-34a in the regenerated bone within the defect area was also higher in irradiated group than non-irradiated group.

Opinions about the role of miR-34a on osteogenesis are conflicting. Fan reported that overexpression of miR-34a significantly increased the osteogenic differentiation of hASCs [14]. Kang showed that miR-34a diminished the inhibitory effect of dexamethasone on osteoblastic differentiation of mMSC [23]. However, Chen demonstrated that miR-34a is a negative regulator of osteoblast differentiation in hMSC [24]. Dang also showed that inhibition of miR-34a suppressed murine arthritis and bone loss [25]. The inconsistent results might be due to the different characteristics of cells and the distinct regulation of osteogenesis under biological or pathological conditions. We found that miR-34a improved the osteogenic differentiation of irradiated BMSCs in vitro. BMSCs that transfected with miR-34a showed more ectopic bone formation when transplanted into nude mice. miR-34a overexpression also enhanced bone regeneration in irradiated tibial defects. These data suggested that miR-34a could promote osteogenesis under irradiated conditions.

Previous studies had verified that NOTCH1 was a direct target for miR-34a by the dual-luciferase reporter assay [20, 26]. The interaction of miR-34a and NOTCH1 plays a vital role in cell proliferation and apoptosis and is associated with development and disease in some tissues [27]. Notch signaling is also important in bone development and regeneration [28]. miR-34a plays a critical role in bone homeostasis, partly through modulating NOTCH1 [14, 29]. Our data demonstrated that irradiation enhanced NOTCH1 expression and decreased osteogenesis in BMSCs. Overexpression of miR-34a in irradiated BMSCs led to decreased NOTCH1 protein expression and increased osteogenesis. Furthermore, this study showed that suppression of NOTCH1 enhanced the expression of RUNX2 and OCN in irradiated BMSCs. Our findings suggested that miR-34a improved the osteogenic differentiation of irradiated BMSCs by suppressing NOTCH1. However, we observed that both of the expression of miR-34a and NOTCH1 were increased in irradiated BMSCs compared with non-irradiated BMSCs. As the interaction of miRNAs with their target genes is dynamic and dependent on many factors [30], it is possibly the availability and abundance of miR-34a that made a difference in the interaction between miR-34a and NOTCH1 expression.

Exploring the delivery systems for miRNAs in bone regeneration has gained increased interests. Different miRNA-regulated systems such as systemic injection [31], site-specific injection [32], and scaffold-based delivery were reported. Hydrogels [33], electrospun nanofibers [34], and nanohydroxyapatite scaffold [35] were used for localized delivery of miRNAs. In this research, we used a collagen-based hydrogel for in situ delivery of miR-34a. This method upregulated the expression of miR-34a within the original bone defects for 2 weeks as demonstrated by intracellular uptake of Cy3-labeled agomir (Additional file 1: Figure S5) and qRT-PCR (Additional file 1: Figure S6). We found that miR-34a enhanced the osteogenic differentiation of BMSCs. However, miR-34a could also influence the function of other cells involved in bone healing and regeneration. miR-34a could inhibit osteoclast differentiation of osteoclast precursors and reduce bone resorption [12, 36]. miR-34a has an anti-angiogenic effect on mouse microvascular endothelial cells [37]. A cell-specific miRNA delivery system is needed in the future research to detect the mechanism of miR-34a on bone regeneration after irradiation in vivo.

Reconstruction of the bone defect after malignant tumor resection is difficult as the healing processes are influenced by the tumor and the treatment strategies including surgery, radiotherapy, or chemotherapy [3]. The regenerative medicine strategies of using stem cells, growth factors, and biomaterials to treat bone defects after tumor resection remain controversial for their potential tumor-promoting effects. miR-34a is a tumor suppressor and regarded as a promising therapeutic agent against cancer [38]. Thus, using miR-34a-targeted therapy to enhance bone regeneration in the bone defect after malignant tumor resection may also suppress tumor recurrence.

## Conclusion

In conclusion, this study demonstrated that miR-34a was upregulated in the process of bone formation after irradiation. Overexpression of miR-34a in radiation-impaired BMSCs could promote the osteogenic ability both in vitro and in vivo. In situ delivery of miR-34a promoted bone regeneration in bone defects of irradiated rat tibias. Our findings identified that miR-34a might be a promising therapeutic target for promoting the reconstruction of the bone defect after malignant tumor resection.

## Additional file

Additional file 1: **Figure S1.** Characterization of BMSCs. **Figure S2.** The miRNA transfection efficiency and effect. **Figure S3.** miR-34a overexpression enhanced the osteoblastic differentiation of 4 Gy irradiated BMSCs in vitro. **Figure S4.** The siRNA transfection efficiency and effect. **Figure S5.** Distribution of agomiR in the bone defect area. **Figure S6.** Expression of miR-34a in the newly formed bone after implantation of miRNA delivery hydrogel. (DOCX 2001 kb)

## Abbreviations

ALP: Alkaline phosphatase
BMSC: Bone marrow mesenchymal stromal cell
COL1: Collagen type I
GAPDH: Glyceraldehyde phosphate dehydrogenase
H&E: Hematoxylin and eosin
miRNA: microRNA
OCN: Osteocalcin
qRT-PCR: Quantitative real-time polymerase chain reaction
RUNX2: Runt-related transcription factor 2
siRNA: Small interfering RNA
