# Supplementary material for: miR-34a promotes bone regeneration in irradiated bone defects by enhancing osteoblastic differentiation of mesenchymal stromal cells in rats
Source: Stem Cell Res Ther. 2019 Jun 18;10:180. doi: 10.1186/s13287-019-1285-y (PMC6582588; doi:10.1186/s13287-019-1285-y)
Supplement: Supplementary file 1 — Figure S1. Characterization of BMSCs. Figure S2. The miRNA transfection efficiency and effect. Figure S3. miR-34a overexpression enhanced the osteoblastic differentiation of 4 Gy irradiated BMSCs in vitro. Figure S4. The siRNA transfection efficiency and effect. Figure S5. Distribution of agomiR in the bone defect area. Figure S6. Expression of miR-34a in the newly formed bone after implantation of miRNA delivery hydrogel. (DOCX 2001 kb) [file 13287_2019_1285_MOESM1_ESM.docx]

**Additional file 1**

**
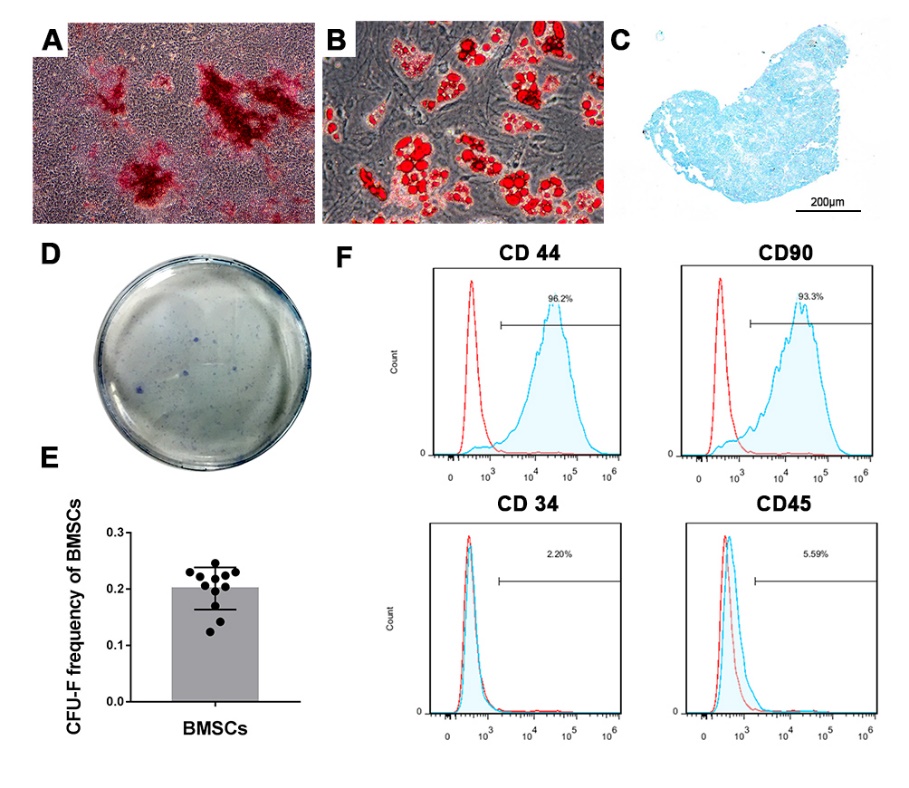
**

**Figure S1. Characterization of BMSCs**

A: Mineral nodes stained with Alizarin Red S (Original magnification ×40) B: Fat droplets stained with Oil Red O (Original magnification ×200). C: Proteoglycans were stained with Alcian blue (scale bar=200μm). D: Representative image of cell colonies. E: The colony frequency of BMSCs (n=4, with three replicates each) F: Flow cytometry analysis of BMSC surface markers.

**
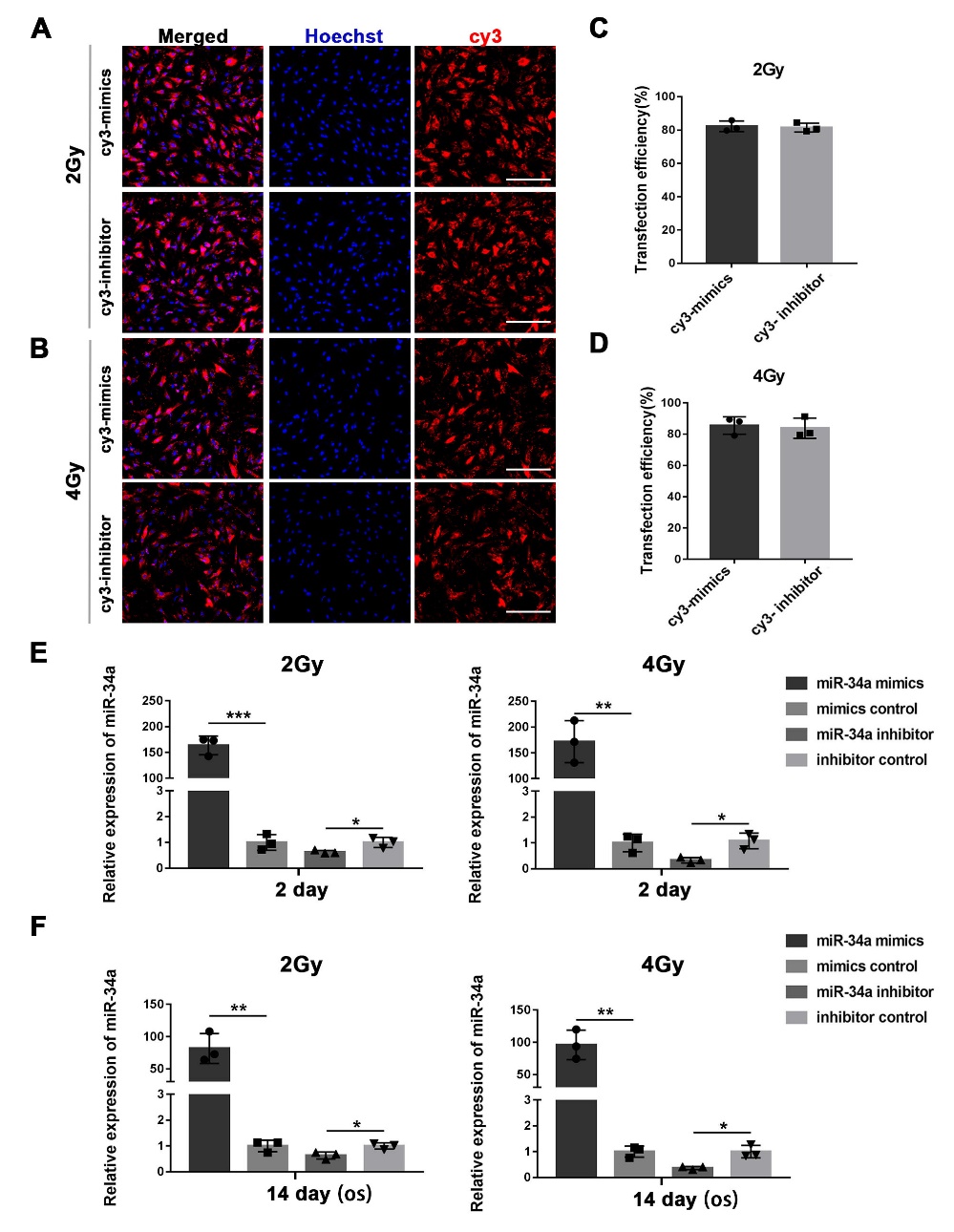
**

**Figure S2.** **The miRNA transfection efficiency and effect**

A, B: The representative images showing the uptake of Cy3-labeled mimics and inhibitor by BMSCs irradiated with 2Gy or 4Gy; scale bar=200μm. C, D: The histograms of Cy3 positive cell percentage. E: miR-34a expression determined by qRT-PCR in the BMSCs 2 days after transfection. F: miR-34a expression determined by qRT-PCR after 14 days of osteoblastic differentiation. Data are shown as mean ± SD, n=3; *p<0.05, **p<0.01, ***p<0.001.


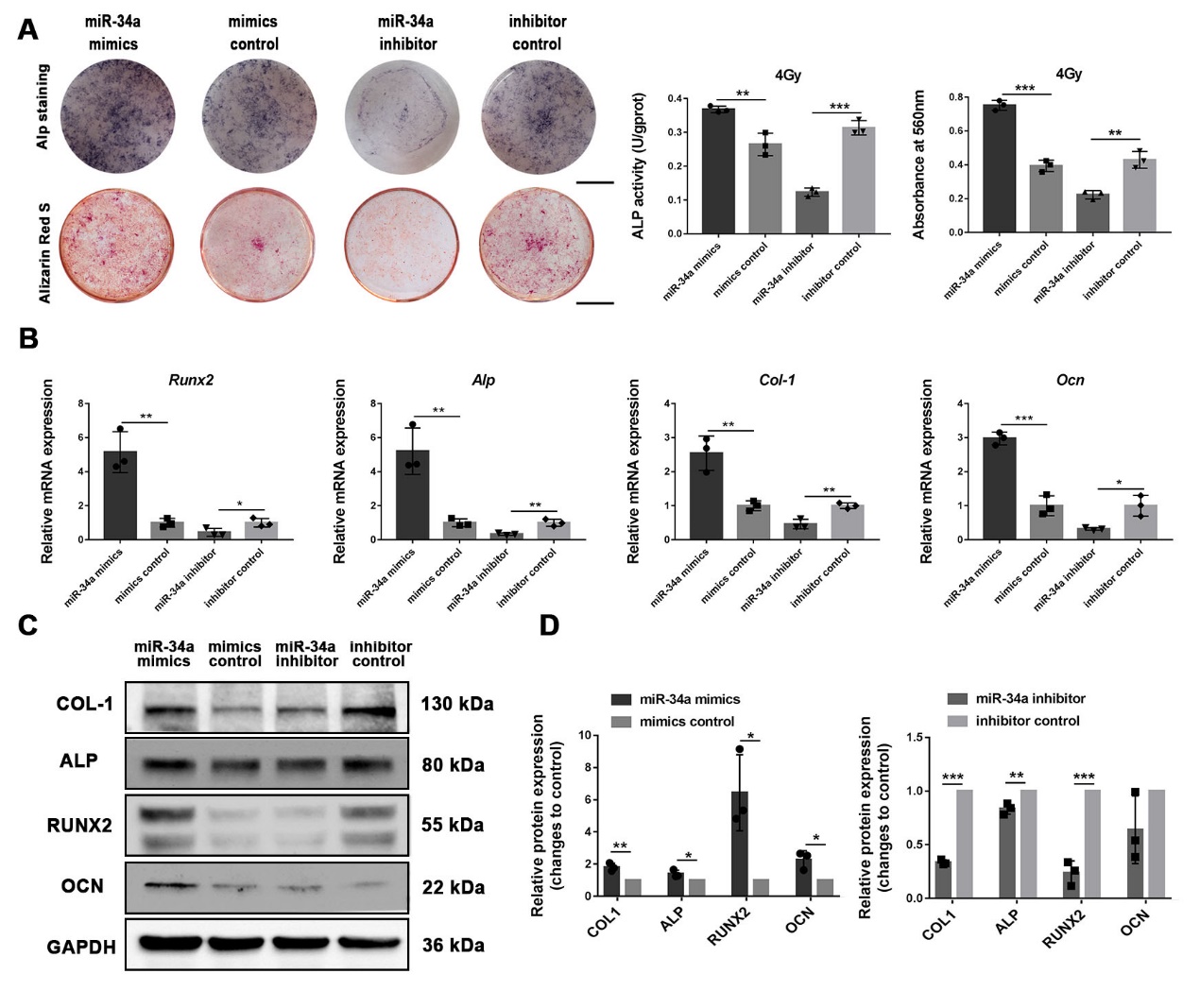


**Figure S3. miR-34a overexpression enhanced the osteoblastic differentiation of 4Gy irradiated BMSCs *in vitro***

A: ALP staining, intracellular ALP activity, Alizarin red staining and the quantitative colorimetric results of Alizarin red staining for BMSCs irradiated with 4Gy after osteogenic induction; scale bar = 1 cm. B: Gene expression of *Runx2*, *Alp*, *Col-1*, and *Ocn* after 14 days of osteogenic induction. C: Western blot analysis of RUNX2, ALP, COL-1, OCN and GAPDH after 14 days of osteogenic induction. D. The quantitative analysis of the western blot results relative to GAPDH (fold to control). Data are shown as mean ± SD, n=3; *p<0.05, **p<0.01, ***p<0.001.

**
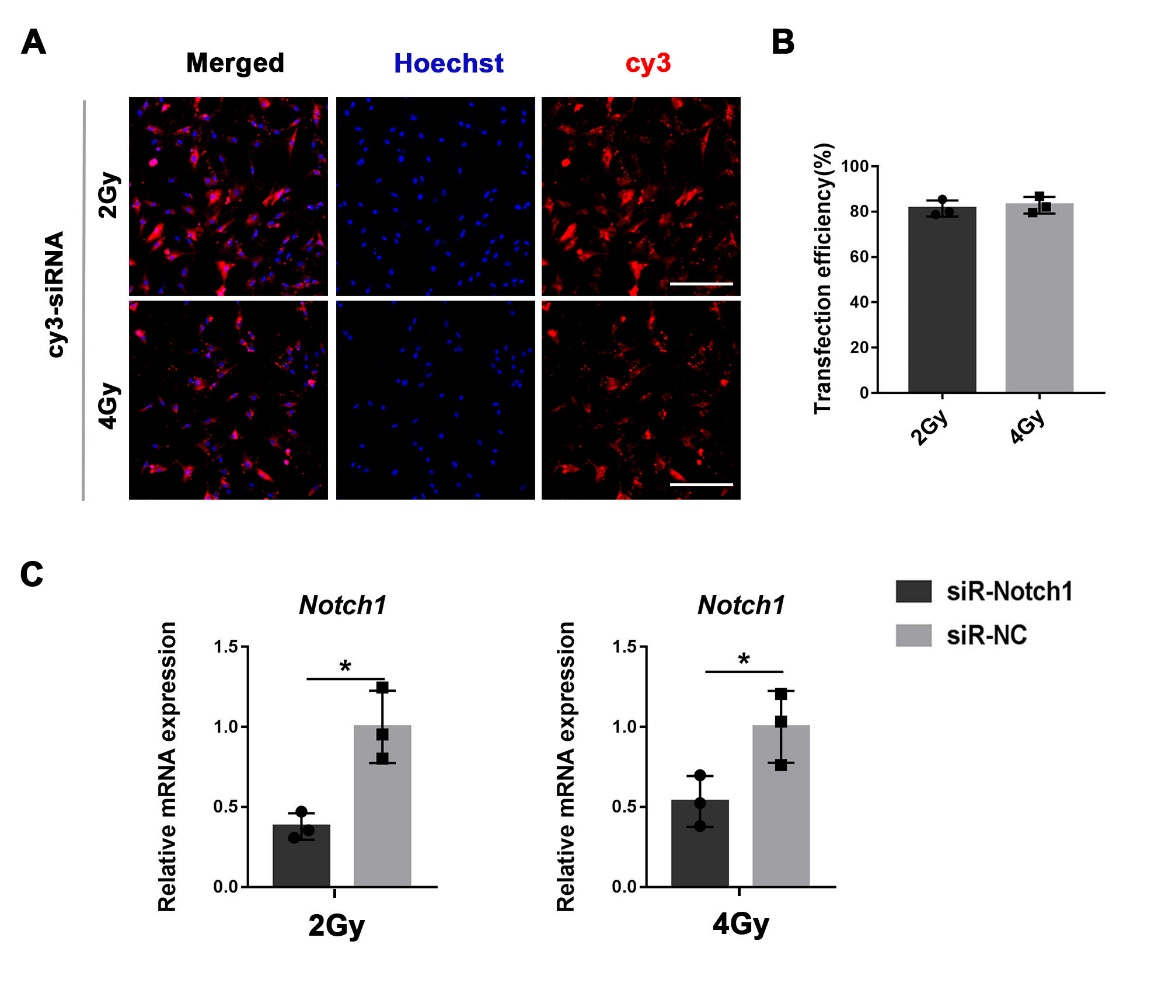
**

**Figure S4. The siRNA transfection efficiency and effect**

A: The representative images showing the uptake of Cy3-siRNA by BMSCs irradiated with 2Gy or 4Gy, scale bar=200μm. B: The histograms of Cy3 positive cell percentage. C: *Notch1* expression determined by qRT-PCR 2 days after transfection. Data are shown as mean ± SD, n=3; *p<0.05.


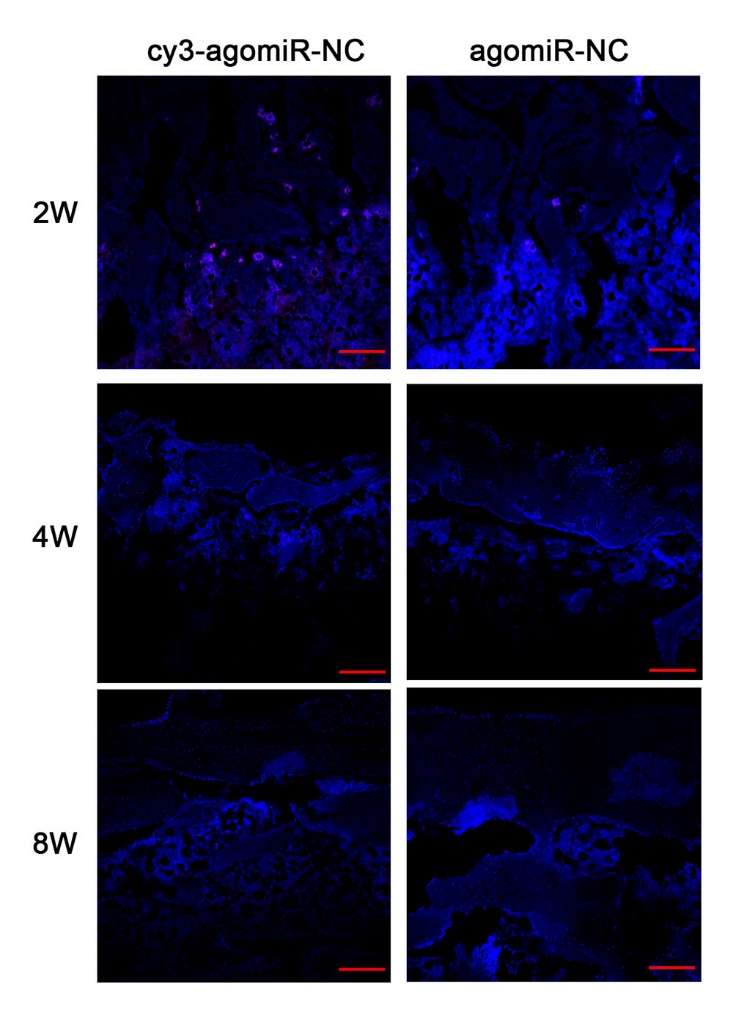


**Figure S5.** **Distribution of agomiR in the bone defect area**

The representative images showing the distribution of cy3-labbeled agomiR NC in the bone defect area 2, 4 and 8 weeks after implantation; scale bar=200μm. agomiR NC was used as control.


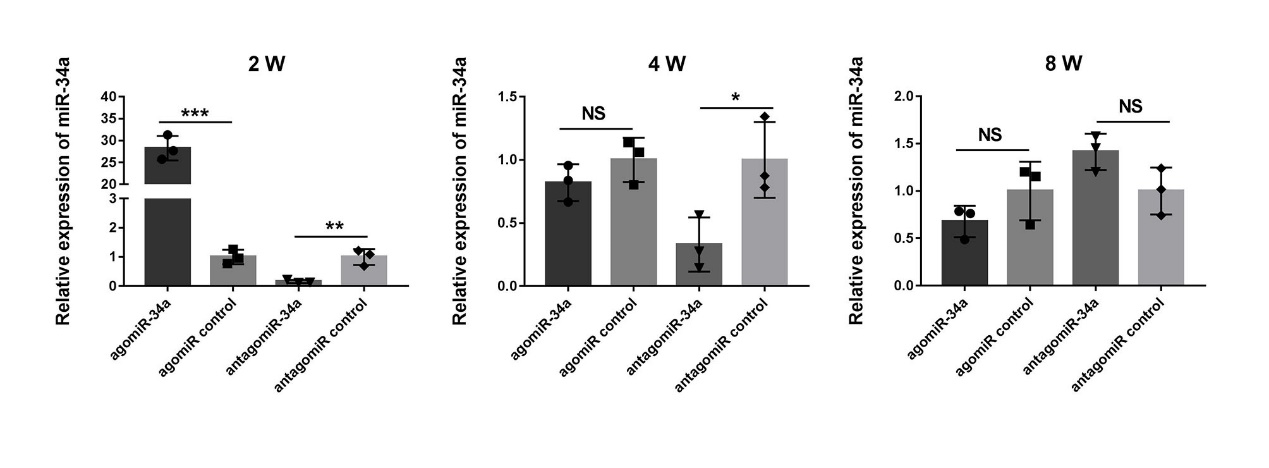


**Figure S6. Expression of miR-34a in the newly formed bone after implantation of miRNA delivery hydroge**l

qRT-PCR analysis of miR-34a expression in the newly formed bone 2, 4 and 8 weeks after implantation. Data are shown as mean ± SD, n=3; *p<0.05, **p<0.01, ***p<0.001.
